# How the risk of suicide and non-suicidal self-injury is assessed, monitored and managed in randomised controlled trials of interventions for youth depression: a scoping review

**DOI:** 10.1136/bmjopen-2025-111993

**Published:** 2026-04-28

**Authors:** Felicity Hudson, Chloe Read-Williams, Grace Williamson, Samantha Lobo, Amelia Hardman, Haleemah Patel, Victoria Pile

**Affiliations:** 1 Department of Psychology, King’s College London, London, UK; 2 King’s Centre for Military Health Research, King’s College London, London, UK

**Keywords:** Risk Assessment, Depression & mood disorders, Adolescent, Clinical trials, Randomized Controlled Trial

## Abstract

**Objectives:**

This scoping review examines randomised controlled trials (RCTs) for young people with depression to explore three key questions: (1) Do RCTs for young people with depression exclude participants based on risk of suicide or self-harm? (2) How is this risk monitored throughout the course of the trial? and (3) When risk is identified, how is this risk managed throughout the RCT?

**Design:**

This project used a scoping review methodology and was conducted in accordance with guidance in the Preferred Reporting Items for Systematic reviews and Meta-Analyses statement.

**Data sources:**

Four electronic databases (PsycINFO, Ovid MEDLINE, PsychARTICLES and Embase) were searched with sets of search terms.

**Eligibility:**

Included studies were RCTs evaluating interventions for depressive symptoms in young people (age 18 or under). Studies published between 1988 and 2025, and in English, were included.

**Data extraction:**

Papers retrieved were independently screened by two reviewers, first by title and abstract and then full text, in accordance with eligibility criteria. Relevant data were then extracted.

**Results:**

89 studies were included. Of these, 55.06% (n=49) excluded young people at screening or baseline stages for risk of suicide or self-harm, with four studies excluding over 10% of participants. Overall, 40.45% (n=36) of studies reported monitoring risk during the trial, with the majority (n=24; 66.67%) using psychometric assessment. Most studies that monitored risk took an active approach to managing it, for example, through referral to external clinical services (n=12; 33.33%) or further clinical involvement from the trial team (n=9; 25%).

**Conclusion:**

To ensure that RCTs for young people with depression are reliable, safe and can be replicated in clinical settings, there needs to be greater clarity on risk procedures in trials and on the expectations of reporting and monitoring the risk of suicide or self-harm.

**Trial registration number:**

The protocol was registered with the Open Science Framework prior to the implementation of the search strategy (https://osf.io/6dz5a; registration DOI: 10.17605/OSF.IO/6DZ5A).

**Strengths and limitations of this study**

- The scoping review methodology is clear and reproducible.
- A comprehensive search and screening process, across a range of databases, was used.
- Wide inclusion criteria were employed to capture a diverse range of studies and interventions.
- Studies not published in English were excluded.
- No formal assessment of study quality or risk of bias was conducted.

## Introduction

 Suicide is a leading cause of death in young people, accounting for over 40 000 deaths worldwide in 2021 in those aged 15–19 years.^1^ A myriad of factors increase the possibility that an individual will attempt to take their own life, with clinical factors such as a mental health diagnosis or a history of non-suicidal self-injury (NSSI; the infliction of minor-to-moderate injuries on the surface of one’s body without suicidal intent)^2^ demonstrating the strongest associations with suicide.^3^ While NSSI and suicidal behaviour are regarded as distinct clinical phenomena, NSSI has been identified as a strong predictor of future suicidal behaviour.^4^ Both suicidality and NSSI are closely linked with depression,^5^ and suicidality is recognised in diagnostic manuals as a symptom of major depressive disorder (MDD) (eg, Diagnostic and Statistical Manual of Mental Disorders, fifth edition).^2^ Depressive symptoms, in turn, predict both suicidal ideation and suicide attempts,^6^ with approximately 38%–50% of adolescents with MDD reporting recurrent suicidal ideation.^7^

Depression is common and impairing in young people, and a longer duration of untreated depression is associated with worse outcomes.^8^ While a number of interventions exist for youth depression, current gold-standard interventions show little benefit over usual care.^9 10^ Consequently, evaluating novel pharmacological and behavioural interventions in RCTs is essential to strengthen the evidence base for treating youth depression. Given the high prevalence of suicidal ideation and NSSI in adolescents with depression,^11 12^ how to effectively manage the risk of suicide and self-harm presents significant ethical and methodological challenges for researchers. For example, whether to include young people presenting with risk of suicide or self-harm in the evaluation of a novel treatment. These challenges are further compounded by the well-documented lack of effective translation of interventions from research to clinical settings. One explanation for this is poor matching between research and clinical populations. For example, excluding young people from the RCT based on their level of risk may limit the generalisability of the findings into clinical settings. This review examines how risk of suicide or self-harm is approached in the design of RCTs for depression in young people and how it is assessed, monitored and managed within these trials.

Currently, there is considerable variability in the ways that risk is both defined and monitored across research trials.^13^ Risk in this context can be defined as the possibility of harm, that is, the suggestion that something may occur without implying that it is probable or will occur.^14^ Despite the evident need for a clear and consistent approach to the risk of suicide and self-harm, it is often unclear what process is taken to assess, monitor and manage risk in RCTs. One approach is to use adverse events (AEs) to monitor risk. Yet, in trials of psychological interventions, the reporting of AEs has been found to be weak or use inappropriate criteria.^15^ For example, a recent systematic review of trials for children and adolescents highlighted that AEs are inconsistently defined across different trials, and the severity of events is rarely documented.^16^ Another approach is to use standardised risk assessments, but there is ongoing debate about their clinical utility, with concerns about the tools currently available.^17^ One advantage of standardised assessment would be to quantify risk in clinical trials, aiding the objective assessment of clinical safety of a new intervention and providing clearer guidelines for who the intervention might benefit. For example, quantification could ensure there is no pattern of increased risk for an individual following a novel intervention. Furthermore, the characterisation of the sample is important, for example, knowledge as to whether the intervention has been evaluated in young people with depression who are experiencing suicidal ideation (and its severity) and what criteria this assessment/judgement is based on. This not only enables comparison across RCTs but also improves matching from research to clinical settings. There is some evidence that standardised instruments, such as the Columbia Suicide Severity Rating Scale (C-SSRS)^18^ and the Scale for Suicidal Ideation,^19^ can elicit valuable information regarding risk. However, the reliability of these scales, as well as their relevance in predicting future suicidal behaviour, has been questioned.^20^

This scoping review aims to understand how risk is approached in terms of inclusion criteria and how risk is assessed and managed in young people (up to 18 years of age) who are receiving psychological and/or pharmacological treatment for depression in RCTs. We explored three research questions:

1. Do RCTs for depression exclude young people based on risk of suicide and self-harm?
2. How is the risk of suicide and self-harm monitored throughout the course of the trial?
3. When the risk of suicide and self-harm is identified, how is it managed in the context of the trial?

Through understanding the variety of approaches to risk of suicide or self-harm in young people with depression, and the assessment tools currently in use, this review aims to highlight future directions for the field and to consider possible approaches to risk management when designing RCTs for this population.

## Methods

### Search strategy

The review was conducted in accordance with guidance in the ‘Preferred Reporting Items for Systematic Reviews and Meta-Analyses’ (PRISMA) statement.^21^ The protocol was registered with Open Science Framework prior to implementation of the search strategy (https://osf.io/6dz5a; registration DOI: 10.17605/OSF.IO/6DZ5A; online supplemental material A).

Four electronic bibliographic databases were searched (PsychINFO, Ovid MEDLINE, PsychARTICLES and Embase) using the database host Ovid from inception to 18 August 2020. The search was rerun on 6 May 2024 and again on 8 December 2025 to identify any new publications since the first search. Databases were searched with the following terms: (depress* OR dysthymi* OR persistent depressive disorder) AND (youth OR young people OR teen* OR adolescen* OR child* OR paediatric OR juvenile OR under 18) AND (RCT OR randomised control* trial OR randomised control* trial). Please see online supplemental material B for the full search strategy.

### Eligibility criteria

The eligibility criteria for included studies were (1) English-language journal articles; (2) human studies; (3) interventions targeting depressive symptoms, including participants seeking help for symptoms of depression/depression diagnosis and/or presenting with depression deemed to exceed a threshold for intervention prespecified by the trial team. Studies where participants had comorbid psychiatric conditions were included with the proviso that the intervention focus was on depression; (4) the focus was to reduce symptoms of depression via intervention; (5) participants were aged 18 years and under; (6) empirical data were collected from a primary source; and (7) the study design was an RCT.

### Study selection and data extraction

Papers retrieved from the initial database search were uploaded to Mendeley. Records were then initially screened by title and then by abstract by two independent researchers in accordance with the research questions and eligibility criteria. Full-text articles were screened by two researchers. Any disagreements were easily resolved through discussion, with none requiring a third-party reviewer. Data from included articles were then extracted. This included bibliographic information (author, title and year), demographics (age range, age mean, number of participants, % female and country), measures (measures used to assess depression, risk assessment used, standardised risk assessment, risk assessment tool, time points of risk assessment, change in risk after/during intervention, study exclusion criteria for harm, further risk assessment within the team and additional assessment or referral risk) and information on the intervention within the RCT (intervention used, psychological and/or pharmacological and/or neurological stimulation therapy).

The purpose of this review was to explore methods of identifying and managing the risk of suicide or self-harm used in a broad range of RCTs. These studies often have different definitions of risk and different approaches to how risk is viewed in the context of the intervention that is being evaluated. In order to summarise and compare studies, information on (1) the reasons that studies excluded participants and (2) how studies managed risk was coded into categories. To address our first question of whether studies excluded participants, reasons for participants being excluded were coded as (1) unspecified suicidal ideation, (2) active suicidal ideation (eg, with intent or a plan), (3) a recent suicide attempt (within 12 months), (4) a history of suicide attempts, (5) NSSI, (6) urgent treatment required (eg, hospitalisation or emergency referral due to risk posed to self or others), (7) clinical questionnaire/screening, (8) unspecified high risk, (9) risk to others and (10) unsafe context (eg, unstable home environment). A number of studies provided multiple reasons for excluding participants and so have been coded into multiple categories. Studies were also coded in terms of risk management over the duration of the trial to address our third question. The study’s approach to risk reported by a participant during the RCT was coded as (1) discontinuation (referring to any studies that removed participants from the RCT when risk was reported), (2) onwards referral or signposting (eg, provision of crisis line contact details or referral to additional clinical services), (3) further clinical involvement from a member of the associated clinical team (eg, study lead or clinical supervisor), (4) not recorded and (5) other. For ease of readability in the Results section, citations will be provided when five or fewer articles are grouped together, but not when six or more articles are grouped.

### Assessment of methodological quality and publication bias

As this is a scoping review, a risk of bias assessment or quality appraisal was not conducted, in line with the Joanna Briggs Institute Manual.^22^ This manuscript was prepared with reference to the PRISMA extension for scoping reviews reporting guidelines and checklist (please refer to online supplemental material C).^23^

### Patient and public involvement

There was no patient or public involvement in this study.

## Results

### Study selection

**Figure 1:**
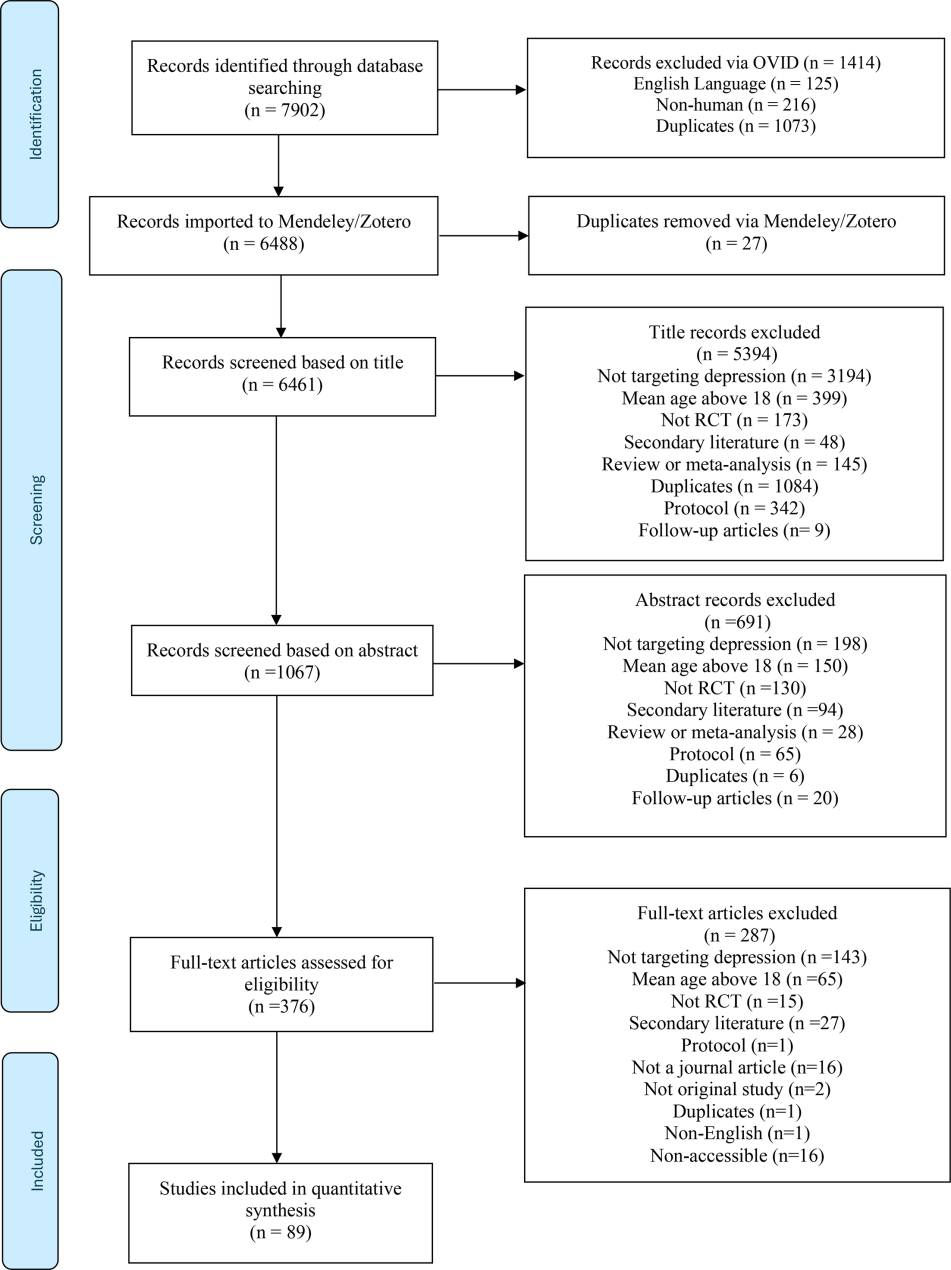
Study selection process (Preferred Reporting Items for Systematic Reviews and Meta-Analyses diagram). RCT, randomised controlled trial.

Please see figure 1 for an illustration of the study selection process. The search identified a total of 7902 studies. 1414 were excluded through Ovid based on the following criteria: 125 were not in English, 216 involved non-human participants and 1073 were duplicates. The remaining 6488 records were exported to a reference manager, which removed an additional 27 duplicates, resulting in 6461 records for title screening. During title screening, 5394 records were excluded for reasons such as duplication, not targeting depression, including participants with a mean age above 18 years, having methodology which was not an RCT, secondary data analysis (and the main RCT paper was included). In the abstract screening stage, 1067 records were screened, with 691 excluded, resulting in 376 records for full-text analysis. At full-text screening, 287 were excluded, resulting in a total of 89 studies meeting inclusion criteria and being included in the synthesis.

### Study characteristics

**Figure 2:**
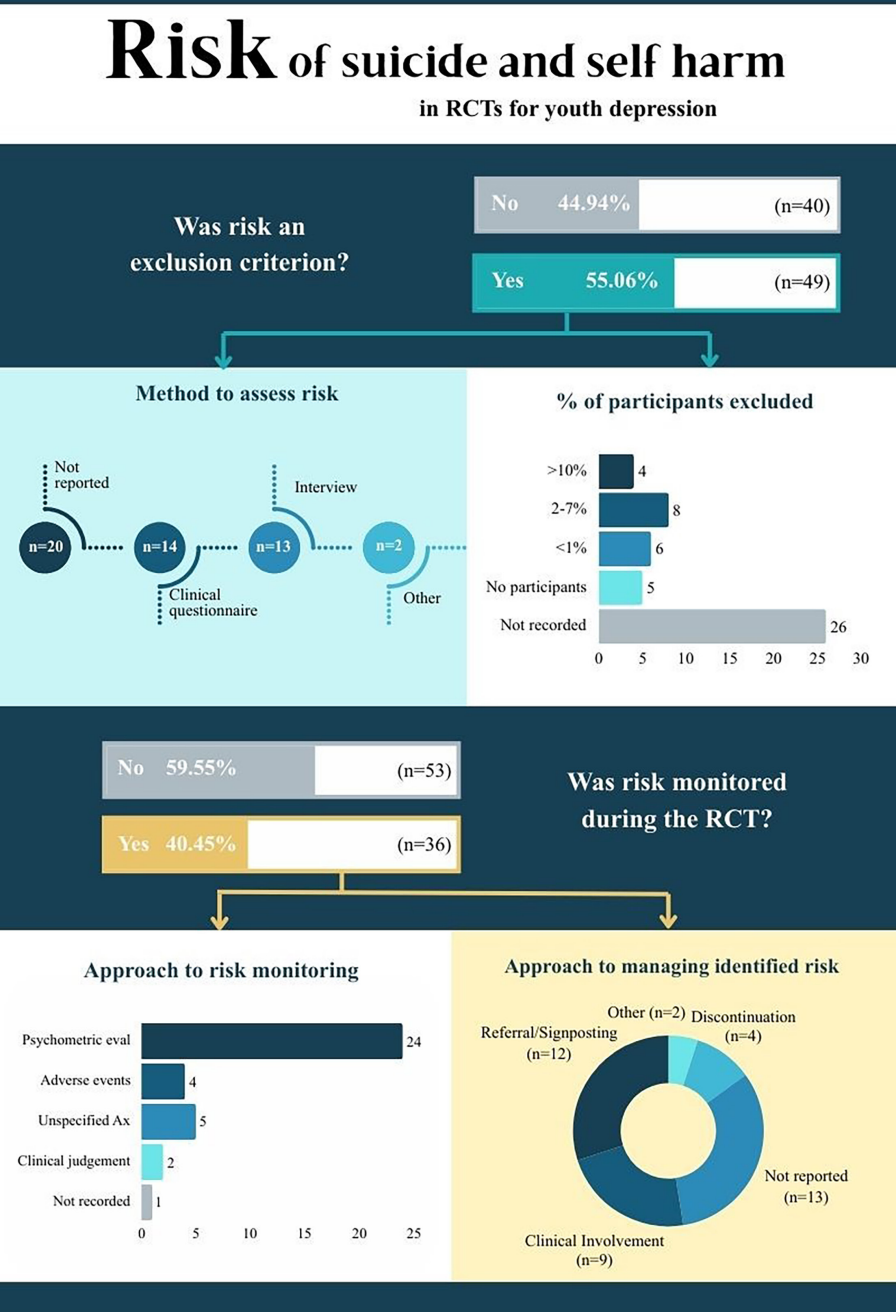
Summary of key findings. RCT, randomised controlled trial.

The characteristics and baseline demographics of the included studies are outlined in online supplemental table 1, and an infographic of the results in figure 2. Study sample sizes ranged from eight participants to 2452^24^ participants. To understand when the majority of included RCTs were published, they were divided into decades. Of the 89 included studies, the majority (73.03%; n=65) were published between 2015 and 2025, 18.18% (n=16) between 2005 and 2014, and 9.09% (n=8) between 1994 and 2004. This suggests there has been an increase in RCTs for depression in youth over time.

Age was reported in 92.13% (n=82) of studies, with seven studies not reporting age. Participants’ mean age varied from 5.21 to 17.06. Of those studies that reported age, the majority focused on young people over age 10: 97.56% (n=80) recorded a mean age between 10 and 17. Only two studies reported mean ages below 10, with one study reporting a mean age of 5.21^25^ and the other a mean age of 9.38.^26^ Gender distribution was reported in 96.63% (n=86) of studies, with the majority (84.88%; n=73) including more female than male participants, 2.33% (n=2) with an equal gender split^27^, and 12.79% (n=11) with more male than female participants (e.g.^28 29^). A total of 7.87% of the 89 studies (n=7) exclusively included a female population (e.g.^30^). Most of the studies (92.13%; n=82) took place in high income (e.g.^31^,^33^) or middle-income (e.g.^34^) countries and, within this, 36 took place in the USA (e.g.^35^,^37^). The remaining seven studies took place in Iran^38^,^40^ (n=3), Uganda^41^ (n=1), Nigeria^42^ (n=1), India^43^ (n=1) and Jordan^44^ (n=1).

A total of 66.29% (n=59) of studies evaluated psychological interventions such as cognitive behavioural therapy (CBT)(e.g.^45^,^50^), interpersonal psychotherapy (e.g.^51^,^53^), behavioural activation (e.g 54,56) or group therapy (e.g.^57 58^). There were 16.85% (n=15) which evaluated pharmacological interventions such as duloxetine, fluoxetine or clomipramine (e.g.^3759^,^62^). A smaller number, 5.62% (n=5), evaluated neurological stimulation therapies such as electroconvulsive therapy^63^ and theta-burst brain stimulation (e.g.^64^). Another 10.11% (n=9) used a combination of psychological and pharmacological interventions (e.g.^65^,^68^), and one study used a combination of neurological stimulation therapies and psychological interventions^69^ . Six of the studies which combined psychological and pharmacological interventions used psychological interventions based on CBT techniques, the exception to this being the three combined psychological and pharmacological interventions published in 2025 (e.g.^70^).

#### Question 1: do RCTs for depression exclude young people based on risk?

A total of 49 (55.06%) studies excluded young people either at the screening or baseline phase for risk. The other studies did not specify risk in their exclusion criteria or in the study procedure.

##### Studies excluding participants for risk of suicide or self-harm

Table 1 outlines the ways that studies identified risk and the criteria that they used to exclude for risk. Out of the 49 papers that excluded individuals due to risk: 20 did not record a criterion or method, 14 studies used clinical questionnaire measures, 13 studies used a form of interview assessment, one used patient history^71^ and one study which was recruiting from hospital admissions excluded young people admitted with suicidality as the primary complaint.^72^

**Table 1:** Reasons and methods for excluding for risk in RCTs of interventions for depression in youth

Author	Year	Exclusion reasons	Exclusion tool	% excluded for risk
Arnott	2015	2, 4, 5	NR	0
Bernal	2019	2	Suicidal Ideation Questionnaire Junior	NR
Bluth	2023	1, 6	Diagnostic Interview Schedule for Children version 4	NR
Bolton	2007	2	NR	NR
Dardas	2025	7	Suicide Intent Scale and Suicide Ideation Scale	NR
De Jonge-Heesen	2020	2	CDI-2	0.31
Diamond	2019	6	Face-to-face clinic visit	NR
Deng	2025	7	Beck’s scale for suicidal ideation	NR
Findling	2020	2, 3	Interview and C-SSRS	NR
Fleming	2012	2	NR	0
Fristad	2016	2	NR	NR
Grudin	2022	2	Unspecified screening and face-to-face assessment	5.19
Gunlicks-Stoessel	2016	2, 10	NR	4.76
Hughes	2013	2, 4	NR	NR
Iftene	2015	2, 9	NR	NR
Ip	2016	6	NR	0
Jones	2021	2, 5	NR	4.06
Kaubisch	2023	2	NR	NR
Keller	2001	2	NR	NR
Lan	2023	3	NR	NR
Lindqvist	2020	2, 4	Online screening and diagnostic interview	6.46
Liu	2025	1	Semi-structured interview	NR
Magantor	2025	6	NR	NR
March	2004	2, 10	Cross-site panel	0.91
McCarty	2013	1	NR	2.44
McCauley	2015	6	NR	0.81
Nelson	2004	1	K-SADS-P	0
O’Dea	2024	2,7	Patient Health Questionnaire for Adolescents and assessment	36.66
Pile	2023	8	Face-to-face assessment	NR
Poppelaars	2016	7	CDI (item 9)	0.21
Ranney	2018	1	Hospitalised with chief complaint of suicidality	19.56
Rohde	2008	8, 9	NR	NR
Saito	2022	1, 3	C-SSRS or history of suicidal ideation/attempt in past year	NR
Sallee	1997	1	Face-to-face assessment	NR
Santomauro	2016	2	Beck Depression Inventory and suicide risk assessment	19
Schniering	2022	2, 5	Semistructured telephone interview	0.95
Shirk	2014	3, 6	History—determined by interview	3.23
Shomaker	2016	2	K-SADS	14.61
Smith	2015	6, 8	NR	6.95
Trowell	2007	6	NR	NR
Upadhyay	2025	1	NR	NR
Vande Voort	2022	2	Judged by the investigator	NR
Wijnhoven	2014	1, 7	CDI	0.36
Wilson	2024	2, 6	K-SADS	3.13
Wright	2017	2	NR	NR
Xu	2025	4	History	NR
Zhang	2025	2	Semi-structured interview	NR
Zheng	2025	2	Semi-structured interview	0
Zsigo	2023	2	Face-to-face assessment	NR

Categories: (1) unspecified suicidal ideation, (2) active suicidal ideation (eg, with intent or a plan), (3) a recent suicide attempt (within 12 months), (4) a history of suicide attempts, (5) NSSI, (6) urgent treatment required (e.g. hospitalisation or emergency referral), (7) clinical questionnaire/screening, (8) unspecified high risk, (9) risk to others and (10) unsafe context (eg, unstable home environment).

CDI-2, CDI second edition; CDI, Children’s Depression Inventory; C-SSRS, Colombia-Suicide Severity Rating Scale; K-SADS, Kiddie Schedule for Affective Disorders and Schizophrenia; K-SADS-P, K-SADS-School Age Children-Present Episode; NR, not recorded.

For the 14 studies that used clinical questionnaires, four specified a cut-off score at which participants were excluded: one specified suicidal ideation alongside a clinical score (>19) on the Child’s Depression Inventory (CDI),^73^ one a score of two on question nine of the CDI (‘I want to end my life’),^74^ one a score of 1 or 2 on items 4 and 5 of the Beck Scale for Suicidal Ideation,^75^ and one a score of 2 or more on the Patient Health Questionnaire-Adolescent item 9 (PHQ-9) (‘thoughts that you would be better off dead, or of hurting yourself in some way’).^76^ The remaining 10 studies using clinical questionnaires reported using these to initially identify possible risk and then assessing the young people further to determine eligibility. For example, in De Jonge-Heesen *et al*, any adolescent reporting a score of two on one item of the CDI-2 (‘I want to end my life’) was approached for a further assessment with a professional in which the presence and severity of suicide risk were checked, and only those deemed at high risk for suicidality after this assessment were excluded.

Studies were categorised in terms of the reasons they gave for excluding participants from the trial, with studies often providing multiple reasons (see table 1). The number of studies per category was (1) unspecified suicidal ideation, n=9; (2) active suicidal ideation (eg, with intent or a plan), n=26; (3) a recent suicide attempt (within 12 months), n=4; (4) a history of suicide attempts, n=4; (5) NSSI, n=3; (6) urgent treatment required (eg, hospitalisation or emergency referral), n=9; (7) clinical questionnaire/screening, n=5; (8) unspecified high risk, n=3; (9) risk to others, n=2 and (10) unsafe context (eg, unstable home environment), n=2. The most common reason for exclusion due to risk was participants exhibiting active suicidal ideation (n=26). Studies included in this category referred to terms such as ‘acute suicidality’,^77^ and risk of suicidality ‘as indicated by clearly stated intent and plans’.^78^ For the urgent treatment category (n=9), this included studies that excluded participants who had previously received emergency treatment and studies where participants were deemed to need support which fell out of the remit of the study.

Of the 49 studies which excluded participants due to risk, 26 (53.06%) did not record how many participants were excluded based on their criteria. For the 23 studies that screened for risk and reported the number of participants excluded due to risk, five studies reported excluding no participants^79^,^83^; six excluded less than 1%; eight studies excluded between 2% and 7% and four excluded more than 10% of participants.^72 76 84 85^

### Question 2: how is risk monitored throughout the course of the trial?

A total of 40.45% (n=36) of papers reported monitoring risk during the course of the trial, outlined in online supplemental table 3. The approaches used were psychometric evaluation, monitoring of AEs and unspecified risk assessments. The majority of these 36 studies (66.67%, n=24) used psychometric evaluation. A range of psychometric evaluation tools was used; the Suicidal Ideation Questionnaire-Junior (SIQ-Jr) was the tool most frequently used, with five studies (13.89%) using it to monitor for risk.^86^,^90^ Four studies used the C-SSRS^7891^,^93^ and two studies used the PHQ-9.^94 95^ Two studies used modified versions of the Mood and Feeling Questionnaire (MFQ): one added a risk question from the PHQ-9 to the end of the short MFQ,^96^ and the other used a modified 15-item version of the MFQ.^97^ Two studies used question nine of the CDI,^98 99^ one used the CDI-2,^74^ and one used the Beck’s Depression Inventory-II.^100^ One study used the Suicide Ideation Assessment Tool^101^ and one used the Suicide Probability Scale and Modified Self-Harm Inventory.^102^

The occurrence of AEs was also used as a tool to monitor risk in four studies. In two studies,^103 104^ the recording of AEs was the only method of monitoring risk, and these studies did not specify how they were recorded or monitored. In two studies,^90 105^ AEs were monitored alongside a psychometric tool.

Five studies described using risk assessments but did not specify what risk assessment was used.^79106^,^109^ One study^110^ did not record how risk was monitored but did specify that risk issues were managed in individual supervision throughout the intervention period, implying that there was a form of risk monitoring being conducted. Two studies mentioned that the clinician would assess risk following a disclosure of risk by a participant.^72 111^

The majority of studies monitored risk at assessment timepoints or otherwise referred to risk as being monitored ‘throughout’.^85^ Seven studies specified that risk was monitored during treatment sessions as well as at baseline and during follow-up assessments.

### Question 3: when risk is identified, how is it managed in the context of the trial?

From the 36 studies which reported monitoring risk during the trial, studies were categorised in terms of how they managed risk over the course of the trial (see online supplemental table 3). Studies were categorised as (1) trial discontinuation (n=4), (2) onwards referral or signposting (ie, provision of crisis line contact details or referral to additional clinical services) (n=12), (3) further clinical involvement (n=9), (4) not recorded (n=13) and (5) other (n=2).

Four studies discontinued the involvement of a participant if risk was identified; three of these studies were pharmaceutical interventions, with two^91 104^ referring to discontinuation after serious AEs related to suicidality or study-related activity, such as side effects of the medication. 12 studies signposted participants to other services or directly referred participants for further support. The format of these referrals varied; for example, one study^81^ provided an ‘emergent intervention’ to offer timely assistance in a crisis while another^97^ coordinated with local Child and Adolescent Mental Health Service teams to refer participants on for further support. Nine studies detailed further clinical involvement after risk was identified, with four of them doing this in conjunction with signposting or onward referrals to other services. 13 studies did not detail how risk was managed once it was identified within the context of the trial. Two studies^88 96^ are classified as ‘other’ as they referred to unspecified safeguarding or risk management procedures. Diamond *et al* recruited young people experiencing suicidal ideation, and so participants and their parents were already receiving psychoeducation on safety planning and suicidal ideation, and suicide severity was already being monitored as primary outcome measures.

For those 36 studies that reported monitoring risk, 23 studies (63.89%) actively reported how many participants were identified as ‘at risk’ over the course of their RCT (online supplemental table 3). Of these 23, five trials did not identify risk in any participants in their trial; nine trials recorded risk in between 1% and 10% of the participants in the study; six trials reported risk from between 12% and 25% of participants, and three trials reported risk from over 50% of participants in the study.^86 92 94^ Some of these studies identified this risk at baseline, such as Bernal *et al*, which found that 52.9% of participants required a further risk assessment after their answers in the SIQ-Jr, and others recorded risk only in relation to new risk that occurred during the trial, such as in the recording of AEs. The remaining 13 studies did not report how many participants were identified as at risk; some of these studies recorded AEs over the trial but without differentiating between risk-related AEs and other AEs (eg, vomiting or nausea in relation to a medication change, or AEs such as medical events unrelated to the trial). As these studies did not provide specific data referring to risk-related events, these were categorised as not recorded.

## Discussion

This scoping review aimed to understand how risk of suicide or self-harm is identified and managed in RCTs for interventions for depression in young people. The review had several key findings; these included that (1) over half of the studies excluded participants from the RCT that were identified as being ‘at risk’; (2) less than half of the studies detailed any form of risk monitoring over the duration of the trial; (3) when risk was monitored, the majority of studies used psychometric assessment tools; and (4) there was significant variation in the ways that studies managed risk once it was identified. This scoping review highlights the need for comprehensive reporting of approaches to identifying and managing risk. A consistent approach to identifying and assessing risk in young people would enable researchers to make informed design decisions regarding risk identification and management in RCTs.

Over half (55.06%) of studies reported excluding young people from the RCT due to the presence of risk. The high presence of suicidal ideation in young people with depression is well-documented in the literature.^7^ The finding that four studies excluded over 10% of those screened illustrates the importance of clearly defining the population that an intervention might benefit and providing reasons for this so that it can be translated to clinical settings. These high exclusion rates also highlight the potential issues when designing exclusion criteria; for example, if this is based on passive suicidal ideation, which is common in young people with depression and considered distinct from suicidal behaviour.^112^ These findings could call into question the representativeness of some study samples (if not clearly defined) and thus the genuine effectiveness of the intervention in young people with depression.

One barrier to the inclusion of participants deemed to be at risk could be the concerns of research ethics committees. For example, Andriessen *et al*^113^ suggest that there could be tensions between the researcher’s priority to establish the effectiveness of an intervention and the committee’s priority to ensure risk is contained and avoided where possible, and this might result in tighter eligibility criteria. It must also be acknowledged that RCT methodology or the treatment being evaluated might be deemed inappropriate for participants at risk and there is often justification for strict exclusion criteria. This distinction can be seen in the three studies which were specifically targeting adolescents with suicidal ideation or intent. Two of the studies^89 101^ did not exclude any participants based on risk but instead specified the risk management procedures in place, while the third^88^ specified an exclusion of those at imminent risk that could not be safely treated on an outpatient basis due to the limitations of the intervention. However, of the studies reviewed here, few stated the reasons for excluding participants for risk-related reasons, and 23.60% (n=21) of studies reported excluding based on risk but did not report the number of young people that were excluded. These omissions present a risk of suicide and self-harm as an optional consideration within the reporting of clinical trial results, rather than a key factor to consider when establishing whether an intervention is safe and suitable for the population. It also makes it more challenging for future studies to replicate or make informed decisions on their own study design as well as the implementation of the intervention in clinical services.

Having adequate risk monitoring in place throughout the course of a research trial is a central expectation. The US National Institute of Mental Health views clinical or safety monitoring of individual participants as one of the four main components of monitoring in clinical RCTs.^114^ According to their guidelines, RCTs for young people with depression would fall into either the ‘Greater than Minimal Risk’ or the ‘Significantly Greater than Minimal Risk’ categories; ‘Greater than Minimal Risk’ includes treatment studies of subjects with serious mental illness and ‘Significantly Greater than Minimal Risk’ refers to clinical trials involving vulnerable populations, including children, or interventions to prevent or treat serious conditions (eg, trials that target suicidal ideation or significant self-harm).^114^ The majority of studies (n=53) did not report whether they monitored risk at all over the course of the study, and a further four referenced risk monitoring but provided no details as to what that monitoring entailed. While this is most likely due to a lack of reporting rather than a lack of clinical care, these omissions result in an absence of direction or guidance for future studies and for what could be required for services implementing the intervention in routine services. The Consolidated Standards for Reporting Trials (CONSORT) provides instruction on the reporting of harms in research on the effects of interventions, and there has been a recent introduction of the CONSORT Harms 2022 which encourages clinical trials to include more detailed information about both the benefits and harms of an intervention.^115^ 30 of the studies were published after this updated guidance, and only 11 provided any detail on how risk was monitored or whether participants were excluded over the course of the trial due to risk of suicide or self-harm. It might be assumed that the majority of studies had some form of AE monitoring. However, even if this is the case, many did not report on the prevalence of AEs and did not explain how events were identified or monitored.

Of the studies that continued to monitor risk throughout the course of the trial, the majority used psychometric assessment tools. However, there was a great deal of variation in the tools that were used to assess risk and in the ways that studies managed any identified risk. Future research would benefit from a consistent approach to using risk assessments, as this would enable researchers to understand the findings and compare the groups as well as speed implementation in clinical settings. For example, the benefits of using similar scales can be seen in three studies.^86 89 90^ These three studies used the SIQ-Jr and, as a result, the suicidality of participants can be compared based on scores on the same questionnaire. While the risk protocol was different for each study, it is possible to understand the levels of risk in each study and how safety procedures were followed to best support the participants. There is an increasingly cautious attitude towards manualised risk assessments in the UK.^17^ Clinical judgement is viewed as the most suitable way of assessing risk and psychometric assessments as a tool to aid this rather than accurate predictive tools in themselves. There has also been a shift away from global stratifications of low/medium/high risk, as a 2023 national enquiry reported that 82% of those who died by suicide had either ‘low’ or ‘no risk’ of suicide in short-term risk assessments.^116^ The changed attitude towards risk assessments comes as part of the growing view that inadequate holistic risk assessments are a significant barrier to effective suicide prevention.^117^ However, relying solely on clinical judgement in research trials is also problematic, as it increases the risk of implicit bias. Pragmatically as well, assessments are also often not conducted by highly experienced clinicians and, given that clinical judgement relies heavily on the clinician’s pre-existing knowledge base and experience,^118^ this further calls into question the reliability of ‘clinical judgement’. There is, therefore, a need for instruments, such as psychometric tools, that are clinically useful and accurately quantify risk of suicide or self-harm. It would clearly be a significant advantage if tools could be developed to accurately predict risk, which they are currently viewed as being unable to do.^119^ As such, it is understandable that current research has not reached a consensus on the most clinically appropriate tool/approach. Currently, the involvement of clinician-delivered risk assessment processes in RCTs is crucial and is something which nine (of 89) studies referred to.^78 79 86 89 92 94 98 110 111^

Overall, this review found that RCTs for young people with depression often do not adequately cater towards the risk of suicide or self-harm that is inherent within this population. Further to this, there is a lack of clarity across trials in how to report risk and many omit reporting risk completely from the manuscripts, leaving risk procedures unclear. These findings highlight the need for a wider consensus on the reporting of risk within trials: a protocol which establishes how trials should define and manage risk based on the population they are targeting, the intervention they are offering and the resources that they have available to them. Not all interventions are clinically appropriate for a population at risk of harm to self, and in many cases, excluding young people who would be better served by other interventions is the safest and most appropriate step. However, the lack of clarity and justification for exclusions in many studies, as well as the lack of consistency across trials, results in risk protocols and appropriate target populations becoming opaque and prevents research from being replicable and effectively implemented in clinical services. These issues create a narrative in which risk is viewed in isolation from a research trial when in fact, in the case of trials for depression where risk is expected, the two are intertwined.

There are a number of limitations to this review which should be considered. The term ‘risk’ is often ill-defined, with many papers failing to mention it despite implying risk protocols were in place through references to AEs and safety procedures. The variation in definitions is understandable given the changes in clinical guidance over the time period included here. For example, the CONSORT Harms 2022 identifies a difference between colloquial and statistical definitions of ‘risk’,^115^ a distinction which is absent from the CONSORT Harms 2004 guidelines.^120^ However, this variance in reporting and definitions meant that some assumptions had to be made in the data extraction and synthesis. A second limitation is that the heterogeneity of research papers identified here made it challenging to group the papers in relation to each research question. While this review did provide a definition of risk as a way of managing the variations in terminology used, it was challenging to ensure that all studies were suitably categorised. Third, the review assessed studies based solely on published manuscript data without checking for registered protocols. Consequently, some risk assessment steps may have been undertaken and reported in online databases but not reported in the final publications. Finally, as this was a scoping review, there was no assessment of methodologies or risk of bias and so we cannot conclude on the quality of the studies. However, the methodological quality is perhaps much less important in this type of review, where we are particularly interested in a specific element of the RCT. Future reviews could, however, explore the relationships between risk reporting/monitoring and quality of study.

Research into interventions for young people with depression is a growing field, and one that has reacted quickly to the increased need in recent years.^121^ As such, this review offers the opportunity to reflect on current practices in the field of young people’s mental health research and question the best ways to evaluate safe and meaningful interventions in the future. There are some pragmatic implications from this review, such as the need to clearly define, operationalise and justify exclusion criteria based on risk, to detail risk monitoring and assessment procedures throughout the course of an RCT, and to have a standardised protocol for managing risk when it occurs. However, this scoping review also highlights areas that require further consideration. For example, it could be useful to explore the process behind exclusion decisions when planning an RCT. This could allow identification of areas in which researchers need more guidance to make informed decisions about their own responsibilities and capacity, as well as how the exclusion criteria map onto routine clinical practice and can be implemented. Further research could also help to identify the nuances of risk definitions and the possibility of a universal definition of risk monitoring and risk assessments to ensure that all clinical research is able to meet appropriate safety standards. This review also highlights a significant disparity in this field of research between high- and low-income countries, with 92.13% of the studies conducted in high-income countries. Globally, RCTs remain less common in low-income countries. Beyond the need to understand how research can be effectively delivered in more resource-constrained environments, it is essential to critically examine how risk assessments can be strengthened across diverse cultural and socioeconomic contexts. Furthermore, current procedures should be evaluated and adapted to ensure that they are contextually appropriate and feasible within these settings.

In summary, this scoping review highlighted important gaps in our shared understanding of what risk is in youth depression (and what depression is without risk) as well as a lack of consistency in how risk is monitored, managed and reported. There has been a growing impetus to address these issues, but currently, there is no consensus on how to quantify and manage the risk of suicide or self-harm. Further research is needed to understand how risk can be operationalised in a helpful and meaningful way in RCTs that can then be translated into routine clinical services.

## Supplementary material

10.1136/bmjopen-2025-111993 online supplemental file 1

10.1136/bmjopen-2025-111993 online supplemental file 2

10.1136/bmjopen-2025-111993 online supplemental file 3

10.1136/bmjopen-2025-111993 online supplemental file 4

10.1136/bmjopen-2025-111993 online supplemental file 5

## Data Availability

All data relevant to the study are included in the article or uploaded as supplementary information.
