# Supplementary material for: How the risk of suicide and non-suicidal self-injury is assessed, monitored and managed in randomised controlled trials of interventions for youth depression: a scoping review
Source: BMJ Open. 2026 Apr 28;16(4):e111993. doi: 10.1136/bmjopen-2025-111993 (PMC13141124; doi:10.1136/bmjopen-2025-111993)
Supplement: online supplemental file 1 [file bmjopen-16-4-s001.docx]

**Open Access Link:** [**OSF Registries | Risk assessment in randomised controlled trials of interventions for youth depression: a scoping review.**](https://osf.io/6dz5a)

**Risk assessment in randomised controlled trials of interventions for youth depression: a scoping review.**

**Main question: In young people (up to 18 years of age) who are receiving psychological or pharmacological treatment for depression in a randomised controlled trial, how is risk (e.g. risk of suicide, self-harm) assessed?**

**Background**

Suicide, the fatal act of self-harm with the intent to end one’s own life (Harding, 2020), is the leading cause of death globally in adolescents aged 15-19 (Asarnow and Mehlum, 2019). Relatedly, the prevalence of non-suicidal self-injury (NSSI) is increasing in adolescence and a major predictor of future death by suicide (Asarnow and Mehlum, 2019). NSSI is defined as the ‘deliberate, self-inflicted destruction of body tissue without suicidal intent and for purposes not socially sanctioned, includes behaviors such as cutting, burning, biting and scratching skin' (“International Society for the Study of Self-injury”, 2018). Mood disorders are a well-documented risk factor for attempting suicide (MacLean, 2011); with the incidence of depression occurring in half to two-thirds of individuals who were successful in their suicide attempts (Harwood et al., 2001). It is thought that approximately 90% of those who commit suicide or die from sudden death seek help from their healthcare system – mostly GPs in the 3 months leading to their death (De Leo et al., 2013), suggesting that primary care can play a major role in the identification and prevention of suicide risk.

For researchers and clinicians, it is crucial to be able to identify risks in adolescents in order to reduce suicide attempts within this population. Instruments of risk assessment have evolved over the years, including clinical professional judgement, actuarial assessment and structured professional judgement. Currently, clinical guidelines in the UK dictate that following self-harm, all individuals should be offered psychosocial assessments (Murphy et al., 2010). This is in order to identify both the current and future risk of harm which is a key component in determining the best possible course of treatment and clinical management. Over time, psychosocial assessments have been developed and refined, yet classically have been designed to be conducted by psychiatrists. Since the 1970’s whereby it became more typical for mental health nurses to carry out risk assessments due to the spike in NSSI rates, a wider range of both clinical and non-clinical staff now conduct risk screenings. Research indicates that non-clinicians and cross-disciplinary clinical staff are as proficient as psychiatrists in carrying out risk assessments with regards to content, comprehensiveness and suitability of referrals for specialised clinical management (Murphy et al, 2010). It is less clear however, what risk assessments are commonly used in research settings and educational settings (which are increasingly the first contact with mental health services in the UK). For example, although greater awareness and understanding of mental health has been garnered in educational professionals, staff often feel unequipped with regards to the best course of action (Evans, 2018). Largely the use of risk assessments examining harm to self and others with adolescents with depression is under-documented, and there is a lack of understanding with regard to models of assessment for youth. Therefore, here we will outline the methods for a systematic scoping review in regard to how risk (to self and others) is assessed in young people with depression. Through understanding the variety of risk assessment tools, we aim to provide guidelines for assessing risk in young people with depression.

**Inclusion Criteria**

The eligibility criteria for included studies are based on the PICOS framework [Methley, 2014].

Population (P): young people aged 18 and under experiencing depression, i.e. they were seeking help for symptoms of depression/depression diagnosis and/or were presenting with depression deemed to exceed a threshold for intervention pre-specified by the trial team. Studies where participants have comorbid psychiatric conditions will be included with the proviso that the intervention focus is on depression. Studies will be included when the reported mean participant age falls below 19 (e.g a mean that falls on 18.5 will be included).

Intervention (I): The trial intervention is designed primarily to decrease symptoms of depression. All treatment interventions for depressive symptoms, e.g psychological or pharmacological, will be eligible. Participants aged under 18 years are the direct recipients of the trial intervention (i.e. interventions targeted at parents will not be included).

Comparators (C): There will be no comparator restrictions.

Outcomes (O): This review plans to give an overview of the current tools and practices that are undertaken within Randomised Control Trials (RCTs) to assess risk to one’s self (e.g., self harm and suicide) and to others and whether there is change in level of risk reported during the trial.

Studies (S): Studies will be eligible if they are RCTs published in English and refer to human subjects. Trials from any country and setting will be eligible, and no restrictions will be set on when the outcome was measured, including follow ups post-treatment.

**Search strategy**

We will conduct a systematic search of four electronic bibliographic databases (PsychINFO, Ovid MEDLINE, PsychARTICLES and Embase) using the database host Ovid. The search strategy applied has been developed collaboratively by three review team members with the overarching aim of conducting a comprehensive search of the relevant literature. To inform development of the search strategy, an informal search was undertaken. Here we examined text words contained in the title, abstract and keyword headings from a selection of relevant literature.

A preliminary search was conducted and analysis of text words contained in the title and abstract of retrieved records was carried out and a second search was undertaken to refine the proposed search terms. Our search will include the following terms: (Depress* OR dysthymi* OR persistent depressive disorder) AND (Youth OR young people OR teen* OR adolescen* OR child* OR paediatric OR juvenile OR under 18) AND (RCT OR Randomised Control* Trial OR Randomized Control* Trial).

Our search will be limited to RCTs which remain the gold-standard methodology for assessing psychological/pharmacological interventions within our chosen population (Emslie, Mayes, & Ruberu, 2005). Furthermore, we have chosen to limit our search to English language articles for feasibility and that pertain to human subjects, in line with our PICOS framework.

This proposed search string was agreed on by three review team authors including a clinical psychologist. Two of the review team authors will perform the search, restricting the search using validated Ovid search filters to English language and human subjects. Additionally, we will utilise the validated search filters allowed by Ovid MEDLINE and PsycINFO to identify RCTs and exclude duplicates from the remaining records using the Ovid de-duplicating function.

**Source of evidence**

*Initial Screening*

Papers retrieved from the initial database search will be uploaded into Mendeley. Records will initially be screened by title and then by abstract by two independent researchers in accordance with the research question and eligibility criteria. Discrepancies in agreement will be reviewed by a third researcher.

*Full-Text Screening*

Any full text articles deemed relevant will be reviewed by two researchers independently. Discrepancies will be discussed with a third reviewer. The final list of articles from the full text screening will be reviewed by a clinical psychologist.

**Data Charting**

A data charting procedure will be applied to all studies included from full-text screening using an adapted charting template from the Joanna Briggs Institute Manual (Aromataris, 2020). The following data will be extracted and charted: publication characteristics (e.g. title, first author, year of publication, area of interest); sample demographics (e.g. sample size, age range, age mean, gender % female, ethnicity, country income level of publication); clinical characteristics (e.g. details of clinical diagnosis, depressive symptoms); measure(/s) of risk assessment (e.g. domains assessed, time points of assessment, number of items in tool, details of psychometric validation of tool; further risk assessment with multidisciplinary team; level of risk at each assessment time point; change in risk assessment outcome; intervention characteristics (e.g. psychological/pharmacological, intervention procedure, mode of delivery, context of delivery, experimental groups); primary outcomes (e.g. measurement instrument, outcome summary). Data charting will be performed by two trained review team members in continuous collaboration and consulting with a third review team member (clinical psychologist).

**Analysis of evidence**

A PRISMA diagram will be made in order to detail the subsequent search strategy numbers at each step of the data collection and analyses. Analysis of data will involve the use of quantitative methods such as counts and frequencies to gain data on study and outcome characteristics (e.g., number of included papers, and the total number of varying risk assessment tools). These will be displayed in a table alongside qualitative data regarding how and when the risk assessment was conducted.

**Risk of bias assessment or quality appraisal**

As this is a scoping review, we will not be conducting a risk of bias assessment or quality appraisal, in line with the Joanna Briggs Institute Manual (Aromataris, 2020).

**Funding**

Dr Victoria Pile is funded by a National Institute for Health Research (NIHR), (Clinical Trials Fellowship) for this research project. This publication presents independent research funded by the National Institute for Health Research (NIHR). The views expressed are those of the author(s) and not necessarily those of the NHS, the NIHR or the Department of Health and Social Care.

**References**

Aromataris E, Munn Z (Editors). JBI Manual for Evidence Synthesis. JBI, 2020. Available from https://synthesismanual.jbi.global. https://doi.org/10.46658/JBIMES-20-01

Asarnow, J.R., & Mehlum, L. (2019). Practitioner Review: Treatment for suicidal and self‐harming adolescents – advances in suicide prevention care. Journal of Child Psychology and Psychiatry, 60(10), 1046-1054 <https://doi.org/10.1111/jcpp.13130>

De Leo, D., Draper, B. M., Snowdon, J., & Kõlves, K. (2013). Contacts with health professionals before suicide: Missed opportunities for prevention?. Comprehensive Psychiatry, 54(7), 1117-1123.

Emslie, G. J., Mayes, T. L., & Ruberu, M. (2005). Continuation and maintenance therapy of early-onset major depressive disorder. Pediatric Drugs, 7(4), 203-217.

Evans R, Parker R, Russell AE, Mathews F, Ford T, Hewitt G, Scourfield J, Janssens A (2018) Adolescent self-harm prevention and intervention in secondary schools: a survey of staff in England and Wales. Child Adolescent Mental Health. <https://doi.org/10.1111/camh.12308>

Harding, M. (2020). Suicide Risk Assessment and Threats of Suicide. Patient. Retrieved 17 August 2020, from https://patient.info/doctor/suicide-risk-assessment-and-threats-of-suicide.

Hawton, K., i Comabella, C. C., Haw, C., & Saunders, K. (2013). Risk factors for suicide in individuals with depression: a systematic review. Journal of affective disorders, 147(1-3), 17-28.

International Society for the Study of Self-injury. (2018, May). What is self-injury? Retrieved from: <https://itriples.org/about-self-injury/what-is-self-injury>.

King, C.A., Grupp‐Phelan, J., Brent, D., Dean, M., Webb, M., Bridge, J.A., … & Rea, M. (2019). Predicting 3‐month risk for adolescent suicide attempts among pediatric emergency department patients. Journal of Child Psychology and Psychiatry, 60, 1055‐ 1064.

MacLean, J., Kinley, D. J., Jacobi, F., Bolton, J. M., & Sareen, J. (2011). The relationship between physical conditions and suicidal behavior among those with mood disorders. Journal of affective disorders, 130(1-2), 245-250.

Methley, A. M., Campbell, S., Chew-Graham, C., McNally, R., & Cheraghi-Sohi, S. (2014). PICO, PICOS and SPIDER: a comparison study of specificity and sensitivity in three search tools for qualitative systematic reviews. BMC health services research, 14(1), 579.

Miranda-Mendizabal, A., Castellvi, P., Pares-Badell, O., Alayo, I., Almenara, J….Alonso, J. (2019). Gender differences in suicidal behavior in adolescents and young adults: systematic review and meta-analysis of longitudinal studies. *International Journal of Public Health, 64*, 265-283. doi:10.1007/s00038-018-1196-1.

Morris R, Kapur N, Byng R; Assessing risk of suicide or self harm in adults. BMJ. 2013 Jul 25347:f4572. doi: 10.1136/bmj.f4572.

Murphy, E., Kapur, N., Webb, R., & Cooper, J. (2011). Risk assessment following self‐harm: comparison of mental health nurses and psychiatrists. *Journal of advanced nursing*, *67*(1), 127-139.

National Confidential Inquiry into Suicide and Homicides by People with Mental Illness, 2012. Annual Report: England, Wales, Scotland, and Northern Ireland. University of Manchester, National Confidential Inquiry into Suicide and Homicide by People with Mental Illness.

Newlove-Delgado T, Ukoumunne OC, Moore D, Ford T, Stein K (2015) Mental health related contact with education professionals in the British Child and Adolescent Mental Health Survey 2004. J Ment Health Train Educ Pract 10(3):159–169. <https://doi.org/10.1108/JMHTEP-02-2015-0007>

Russell, E.A., Heron, J., Gunnell, D., Ford, T., Hemani, G., Joinson, C., … & Mars, B. (2019). Pathways between early life adversity and adolescent self‐harm: The mediating role of inflammation in the Avon Longitudinal Study of Parents and Children (ALSPAC). *Journal of Child Psychology and Psychiatry*, 60, 1094‐ 1103.

*Self-harm, suicide and risk: a summary*. Rcpsych.ac.uk. (2010). Retrieved 17 August 2020, from <https://www.rcpsych.ac.uk/pdf/ps03-2010x.pdf>.

Shain, B. (2016). Suicide and suicide attempts in adolescents. *Pediatrics*, *138*(1).

Zelazny, J., Melhem, N., Porta, G., Biernesser, C., Keilp, J.G., Mann, J.J., Oquendo, M.A., Stanley, B., & Brent, D.A. (2019). Childhood maltreatment, neuropsychological function and suicidal behavior. *Journal of Child Psychology and Psychiatry*, 60, 1085‐ 1093.

Webster, C.D., Nicholls, T.L., Martin, M.L., Desmarais, S.L., & Brink, J. (2006). Short-Term Assessment of Risk and Treatability (START): The Case for a New Structured Professional Judgment Scheme, Behavioural Sciences and the Law 24, 747-766. DOI: 10.1002/bsl.737
