# Supplementary material for: How the risk of suicide and non-suicidal self-injury is assessed, monitored and managed in randomised controlled trials of interventions for youth depression: a scoping review
Source: BMJ Open. 2026 Apr 28;16(4):e111993. doi: 10.1136/bmjopen-2025-111993 (PMC13141124; doi:10.1136/bmjopen-2025-111993)
Supplement: online supplemental file 2 [file bmjopen-16-4-s002.docx]

**Supplementary File B: Search Strategy**

The literature’s searching strategies used in OVID. Databases searched- Medline, APA PsychInfo, APA PsychArticles, Embase

| **No** | **Searches** |
| --- | --- |
| #1 | depress* [title/abstract/keyword] |
| #2 | dysthymi* [title/abstract/keyword] |
| #3 | persistent depressive disorder [title/abstract/keyword] |
| #4 | #1 OR #2 OR #3 |
| #5 | youth [title/abstract/keyword] |
| #6 | young people [title/abstract/keyword] |
| #7 | teen* [title/abstract/keyword] |
| #8 | adolescen* [title/abstract/keyword] |
| #9 | child* [title/abstract/keyword] |
| #10 | paediatric [title/abstract/keyword] |
| #11 | juvenile [title/abstract/keyword] |
| #12 | under 18 [title/abstract/keyword] |
| #13 | #5 OR #6 OR #7 OR #8 OR #9 OR #10 OR #11 OR #12 |
| #14 | RCT [title/abstract/keyword] |
| #15 | randomised control* trial [title/abstract/keyword] |
| #16 | randomized control* trial [title/abstract/keyword] |
| #17 | #14 OR #15 OR #16 |
| #18 | #4 AND #13 AND #17 |
| #19 | limit 18 to english language |
| #20 | limit 19 to humans |
| #21 | remove duplicates from #20 |
|  | **Records imported to reference manager** |
