# Supplementary material for: How the risk of suicide and non-suicidal self-injury is assessed, monitored and managed in randomised controlled trials of interventions for youth depression: a scoping review
Source: BMJ Open. 2026 Apr 28;16(4):e111993. doi: 10.1136/bmjopen-2025-111993 (PMC13141124; doi:10.1136/bmjopen-2025-111993)
Supplement: online supplemental file 4 [file bmjopen-16-4-s004.docx]

**Table 1.** Characteristics of RCTs of interventions for depression in youth

| Author | Year | Target population | Mean age | Sample size | % Female | Country | Details of intervention |
| --- | --- | --- | --- | --- | --- | --- | --- |
| Arnott [26] | 2020 | Overweight/obese adolescents meeting criteria for MDD or DD-NOS | 14.27 | 8 | 50 | England | BA sessions |
| Bernal [27] | 2019 | Adolescents with MDD | NR | 121 | 52.90 | Puerto Rico | CBT and TEPSI |
| Birmaher [28] | 1998 | Hospitalised adolescents with treatment-resistant MDD | 16.2 | 27 | 70.37 | USA | Amitriptyline |
| Bluth [29] | 2023 | Adolescents with subsyndromal depressive symptoms | 15.81 | 59 | 70 | USA | MSC-T |
| Bohr [30] | 2023 | Inuit youth exhibiting low mood, negative affect, depressive presentations, or significant levels of stress | NR | 24 | NR | Canada | SPARX |
| Bolton [31] | 2007 | Adolescent survivors of war and displacement with high depressive symptoms | 15 | 314 | 57.30 | Uganda | IPT-G |
| Brent [32] | 2008 | Adolescents with SSRI-resistant depression | 15.9 | 334 | 69.70 | USA | SSRI and CBT |
| Carter [33] | 2015 | Adolescents receiving treatment from a health or social care professional for depression | 15.4 | 87 | 78.16 | England | Preferred Intensity Exercise Intervention |
| Dardas [34 | 2025 | Adolescents with mild to moderate depression | 16.1 | 109 | 52.78 | Jordan | Culturally adapted digital health intervention |
| De Jonge-Heesen [35] | 2020 | Adolescents at high-risk for depression | 13.59 | 130 | 63.80 | The Netherlands | CBT depression prevention program – Op Volle Kracht 2.0 |
| DelBello [36] | 2014 | Adolescents with moderate to severe MDD | 14.8 | 308 | 64 | USA | STS |
| Deng [37] | 2025 | Adolescents with first-episode MDD | 15.3 | 40 | 67.5 | China | Adjunctive tDCS |
| Diamond [38] | 2019 | Adolescents with clinically significant levels of suicidal ideation and moderate depressive symptoms | 14.87 | 129 | 81.90 | USA | ABFT |
| Diamond [39] | 2010 | Adolescents with clinically significant levels of suicidal ideation and moderate depressive symptoms | 15.1 | 66 | 83 | USA | ABFT |
| Dietz [40] | 2015 | Preadolescents diagnosed with a current depressive disorder (MDD, dysthymia, DD-NOS) | 10.75 | 42 | 66.60 | Canada | FB-IPT |
| Ding [41] | 2025 | Hospitalised adolescents meeting ICD-10 criteria for depression | 16.34 | 100 | 55 | China | Oral bifidobacterium and high frequency repetitive  transcranial magnetic stimulation |
| Do [42] | 2021 | Adolescents with mild depression | 15.71 | 376 | 53.72 | South Korea | Computer-based CBT – Hangbok-Nuri program |
| Dobson [43] | 2010 | Adolescents at high-risk of depression | 15.26 | 46 | 69.60 | Canada | CBT |
| Du [44] | 2025 | Hospitalised adolescents with MDD diagnosis and suicidal ideation | 15.42 | 143 | 67.13 | China | Age dose ECT |
| Findling [45] | 2020 | Children and adolescents with MDD | 13 | 473 | 60 | USA and Canada | Vilazodone |
| Fleming [46] | 2012 | Adolescents excluded from mainstream education, with possible through to almost certain depressive disorder | 14.9 | 32 | 44 | New Zealand | Computerised-CBT – SPARX |
| Fristad [47] | 2016 | Youth with MD, dysthymia, or DD-NOS. | 11.6 | 72 | 43 | USA | Omega-3 fatty acids and PEP |
| Gaete [48] | 2016 | Adolescents at risk for depression | 15.9 | 342 | 50.29 | Chile | CBT-based Program – YPSA-R |
| Garber [49] | 2009 | Adolescents at risk for depression | 14.8 | 316 | 58.50 | USA | CB Prevention Program |
| Gearing [50] | 2020 | Adolescents with a depressive disorder or depressive symptoms | 14.65? | 20 | 80 | USA | Tech Connect – between-session SMS messages or calls |
| Goodyer [10] | 2004 | Adolescents with unipolar MDD | 15 | 470 | 74.90 | England | CBT and short-term psychoanalytical therapy |
| Grudin [51] | 2022 | Adolescents with mild-to-moderate MD | 15.4 | 32 | 59 | Sweden | Therapist-guided and self-guided I-BA |
| Gunlicks-Stoessel [52] | 2016 | Adolescents diagnosed with MDD, dysthymic disorder, DD-NOS, or adjustment disorder with depressed mood | 15.2 | 15 | 86.67 | USA | IPT-AP |
| Herrera [53] | 2025 | Adolescents hospitalized with MDD | 15 | 64 | 82.81 | USA | Adjunctive Triple Chronotherapy |
| Hughes [54] | 2013 | Nonmedicated adolescents with MDD | 17 | 30 | 33 | USA | Aerobic exercise |
| Iftene [55] | 2015 | Youth with MDD | 15.25 | 88 | 55.70 | Romania | Group REBT/CBT and sertraline |
| Ip [56] | 2016 | Adolescents with mild-to-moderate depression | 14.64 | 257 | 68.10 | Hong Kong | Internet-based depression prevent program – Grasp the Opportunity |
| Jones [57] | 2021 | Adolescents with elevated depressive symptoms, who did not yet have depressive disorders | 14.01 | 186 | 66.70 | USA | IPT-AST |
| Kaubish [58] | 2023 | Youth with current or remitted MDD | 15.73 | 77 | 80.50 | Germany | Web-based PPI |
| Keller [59] | 2001 | Adolescents with MD | 14.9 | 275 | 62.18 | USA and Canada | Paroxetine |
| Kitchen [60] | 2020 | Young people meeting criteria for MDD | 15.65 | 22 | 81.82 | England | BA |
| Kosik-Gonzalez [61] | 2025 | Adolescents with MDD experiencing current suicidal thinking with intent | 14.9 | 146 | 78.1 | USA, Spain, France, Brazil, Italy, Hungary, Poland | Esketamine nasal spray and oral midazolam |
| Kye [62] | 1996 | Adolescents with MDD | 14.85 | 31 | 29.03 | USA | Amitriptyline |
| Lan [63] | 2023 | Adolescents with MDD and suicidal ideation | 14.8 | 51 | 88.24 | China | Esketamine |
| Li [64] | 2022 | Adolescents with subthreshold depression | 15.07 | 29 | 82.76 | China | LBP |
| Li [65] | 2024 | Adolescents with subthreshold depression | 15.13 | 29 | 79.17 | China | LBP |
| Libuda [66] | 2020 | Hospitalised adolescents with vitamin D deficiency and at least mild depression | 15.95 | 113 | 75.22 | Germany | Oral Vitamin D₃ supplementation |
| Lindqvist [67] | 2020 | Adolescents meeting criteria for MDD | 16.55 | 76 | 80 | Sweden | IPDT |
| Liu [68] | 2025 | Adolescents with non–treatment-resistant MDD | 14.35 | 74 | 79.73 | China | Accelerated intermittent theta burst stimulation |
| Liu [69] | 2025 | Young people hospitalised with MDD and self-harm behaviour | 15.2 | 160 | 58.13 | China | Real-time EEG-triggered repetitive TMS and group therapy (CBT and DBT) |
| Luby [24] | 2018 | Children with early-onset depression | 5.21 | 229 | 34.93 | USA | PCIT-ED |
| Lynch [70] | 2011 | Adolescents with SSRI-resistant depression | 15.9 | 334 | 69.76 | USA | CBT and antidepressant medication switch |
| Magantor [71] | 2025 | Adolescents with at least moderate depressive symptoms | 15.6 | 175 | 61.7 | UAE | Culturally adapted CBT |
| March [72] | 2004 | Adolescents diagnosed with MDD | 14.6 | 439 | 54.40 | USA | Fluoxetine and CBT |
| McCarty [73] | 2013 | Early adolescents with elevated depressive symptoms who did not yet have depressive disorders | 12.75 | 120 | 60 | USA | PTA |
| McCauley [74] | 2015 | Adolescents with a diagnosis of MD, DD-NOS, or Dysthymia | 14.9 | 60 | 64 | USA | A-BAP |
| Moeini [75] | 2019 | Female adolescents with mild to moderate depressive symptoms | 16.35 | 128 | 100 | Iran | Web-based intervention – DAD course |
| Nelson [76] | 2004 | Children meeting DSM-IV criteria for depression | 10.3 | 28 | 28.57 | USA | CBT through videoconferencing |
| Nicol [77] | 2022 | Adolescents newly diagnosed with comorbid depression and anxiety | 14.7 | 17 | 88.24 | USA | Chatbot-Delivered CBT |
| O’Dea [78] | 2020 | Adolescents living in Australia | 14.82 | 193 | 86.50 | Australia | WeClick app |
| O’Dea [79] | 2024 | Adolescents with mild to moderate depressive symptoms | 15.89 | 569 | 74.2 | Australia | Self-directed and nonsequential CBT-based smartphone app |
| Petty [25] | 2009 | Overweight, sedentary children | 9.38 | 207 | 42 | USA | Aerobic exercise program – low or high dose |
| Pile [80] | 2021 | Adolescents scoring above cut-off for depression on the MFQ | 17.06 | 56 | 60.70 | England | ICBI |
| Poppelaars [81] | 2016 | Adolescent females with subclinical depression | 13.35 | 208 | 100 | The Netherlands | CBT Programs – SPARX and OVK |
| Ranney [82] | 2018 | Adolescents presenting to the emergency department, who reported past-year physical peer violence and current depressive symptoms | NR | 116 | 58 | USA | iDOVE |
| Roberts [83] | 2003 | Children with elevated depressive symptoms | 11.89 | 189 | 49.74 | Australia | Depression Prevention Program |
| Rohde [84] | 2008 | Adolescents with MDD | 14.6 | 242 | 57.90 | USA | Fluoxetine and CBT |
| Saito [85] | 2022 | Children and adolescents with MDD | 14.5 | 149 | 62.84 | Japan | Duloxetine |
| Sallee [86] | 1997 | Nonsuicidal outpatient adolescents meeting criteria for MD | 16.2 | 16 | 31.25 | USA | Clomipramine |
| Sanchez- Hernandez [87] | 2019 | Youth with elevated depressive symptoms | 13.88 | 89 | 51.70 | Spain | The Smile Program |
| Santomauro [88] | 2016 | Adolescents with ASD and reporting symptoms in the range of mild depression | 15.75 | 20 | 40 | Australia | CB |
| Schleider [89] | 2021 | Adolescents with elevated depression symptoms | NR | 2452 | 88.09 | USA | BA-SSI and GM-SSI |
| Schniering [90] | 2022 | Adolescents with comorbid anxiety and depressive disorder | 14.29 | 91 | 66 | Australia | Internet based CP |
| Seddigh [91] | 2023 | Adolescent females with elevated depressive symptoms | 14.07 | 62 | 100 | Iran | Yoga Therapy |
| Shirk [92] | 2014 | Adolescents with a depressive disorder and a history of interpersonal trauma | 15.48 | 43 | 83.72 | USA | m-CBT |
| Shomaker [93] | 2016 | Overweight/obese adolescent females with mild-to-moderate depressive symptoms | 15.05 | 119 | 100 | USA | CB |
| Shomaker [94] | 2017 | Overweight/obese adolescent females with mild-to-moderate depressive symptoms | 14.99 | 33 | 100 | USA | Mindfulness-based program |
| Smith [95] | 2015 | Adolescents with elevated depressive symptoms | NR | 112 | NR | England | C-CBT – Stressbusters |
| Taghvaienia [96] | 2020 | High school female students with moderate/mild depression | 16.99 | 60 | 100 | Iran | PI |
| Trowell [97] | 2007 | Children and young adolescents with moderate to severe depression | 12 | 72 | 38 | England, Greece and Finland | Individual Therapy (FIPP) and Family Therapy (SIFT) |
| Upadhyay [98] | 2025 | Adolescents with MDD | 14 | 32 | 53.13 | India | tDCS |
| Vande Voort [99] | 2022 | Adolescents with moderate to severe MDD | 15.4 | 176 | 78.06 | USA | Pharmacogenetics Testing results available |
| Wang [100] | 2025 | Adolescents with treatment-resistant MDD | NR | 120 | 79.17 | China | Magnetic seizure therapy and modified ECT |
| Weisz [101] | 2009 | Youths with MDD, dysthymic disorder, or minor depressive disorder | 11.77 | 57 | 56 | USA | CBT |
| Wijnhoven [102] | 2014 | Adolescent females with elevated depressive symptoms | 13.3 | 102 | 100 | The Netherlands | CBT component of OVP |
| Wilson [103] | 2024 | Adolescents seeking help for low mood | 14.75 | 16 | 75 | UK | Interpersonal counselling |
| Wolff [104] | 2020 | Children meeting criteria for a depressive disorder (MDD and/or dysthymia) and a conduct problem disorder (ODD and/or conduct disorder) | 11.55 | 30 | 66 | USA | DR |
| Wright [105] | 2017 | Adolescents with low mood/depression, reporting symptoms in line with a depressive disorder | 15.35 | 91 | 65.93 | England | CCBT – Stressbusters |
| Xu [106] | 2025 | Hospitalised adolescents with MDD | 15.1 | 73 | 55 | China | Escitalopram combined with short-term trauma stabilization techniques |
| Yang [107] | 2016 | Adolescents with MDD | 14.96 | 45 | 55.56 | USA | ABM |
| Yi [108] | 2024 | Children who survived abduction in Nigeria reporting signs of depression | NR | 450 | 51.56 | Nigeria | CBT, art therapy and music therapy |
| Zhang [109] | 2025 | Adolescent outpatients with depressive disorder | 15.22 | 93 | NR | China | Sertraline combined with psychological hotline-based Naikan therapy |
| Zheng [110] | 2025 | Adolescents with mild to severe depression | 15.07 | 30 | 76.67 | China | EMDR and SSRI |
| Zsigo [111] | 2023 | Adolescents with MD | 15.35 | 71 | 81.43 | Germany | Task-based ER training |

A-BAP- Adolescent Behavioral Activation Program, ABFT- Attachment-Based Family Therapy, ABM- Attention Bias Modification, ASD – Autism Spectrum Disorder, BA- Behavioural Activation, BA-SSI – Behavioral Activation Single Session Intervention, BDI – Beck Depression Inventory, CB – Cognitive Behavioural, CBT- Cognitive Behavioural Therapy, C-CBT- Computerised Cognitive Behavioural Therapy, CCT- Child Centered Therapy, CDI – Children’s Depression Inventory, CP – Chilled Plus, DAD- Dorehye Amozeshie Dokhtaran, DBT- Dialectical Behavior Therapy, DD-NOS – Depressive Disorder Not Otherwise Specified, DR- Decision Rule-based treatment, DSM-IV criteria – Diagnostic and Statistical Manual of Mental Disorders (4^th^ edition), EBP-D- Evidence-based Practice for Depression, ECT - Electroconvulsive Therapy, EEG- Electroencephalogram, EMDR- Eye Movement Desensitisation and Reprocessing, ER – Emotion Regulation, EUC- Enhanced Usual Care, FB-IPT – Family-based interpersonal psychotherapy, FLX- Fluoxetine, FPIP- Individual Therapy, GM-SSI- Growth Mindset Single Session Intervention, HE- Health Education, I-BA- Internet-delivered behavioural activation, ICBI – Imagery-based cognitive behavioural intervention, ICD-10 - International Classification of Disease-10, IPDT – Affect-focussed Psychodynamic Internet-Based Therapy, IPT-A – Interpersonal Psychotherapy for Depressed Adolescents, IPT-AP- Interpersonal Psychotherapy for Depressed Adolescents and Parents, IPT-AST – Interpersonal Psychotherapy-Adolescent Skills Training, IPT-G - Interpersonal Therapy for Groups, LBP – Lycium barbarum polysaccharide, MDD – Major Depressive Disorder, m-CBT- Modified Cognitive Behavioral Therapy, MD – Major Depression, MSC-T – Mindful Self-Compassion for Teens, NDST- Non-directive Supportive Therapy, ODD – Oppositional Defiant Disorder, OVP – Op Volle Kracht, PCIT-ED – Parent-Child Interaction therapy targeting emotion development, PEP- individual-family psychoeducational psychotherapy, PI – Positive Intervention, PPI – Positive Psychology Intervention, PTA – Positive Thoughts and Actions, REBT/CBT-Rational-Emotive and Cognitive-Behavior Therapy, SEQ- Sequential Treatment, SIFT- Family Therapy, SPARX – Smart, Positive, Active, Realistic, X-Factor thoughts, SSI- Single Session Intervention, SSRI- Selective Serotonin Reuptake Inhibitor, STS- Selegine transdermal patches, TAU- Treatment as Usual, TCT – Adjunctive Triple Chronotherapy, tDCS- Transcranial Direct Current Stimulation, TEPSI- Talleres de Educación Psicológica, TMS - Transcranial Magnetic Stimulation.
