# Supplementary material for: How the risk of suicide and non-suicidal self-injury is assessed, monitored and managed in randomised controlled trials of interventions for youth depression: a scoping review
Source: BMJ Open. 2026 Apr 28;16(4):e111993. doi: 10.1136/bmjopen-2025-111993 (PMC13141124; doi:10.1136/bmjopen-2025-111993)
Supplement: online supplemental file 5 [file bmjopen-16-4-s005.docx]

**Table 3.** Methods of monitoring risk in RCTs of interventions for depression in youth

| Author | Year | Risk Tool | Time Point | How risk was managed | % identified as at risk |
| --- | --- | --- | --- | --- | --- |
| Arnott | 2020 | Unspecified risk assessment | NR | 3 | 0 |
| Bernal | 2019 | SIQ-Jr | Before intervention | 3 | 52.9 |
| Bohr | 2023 | Clinical judgement | Pre and post intervention | 3 | NR |
| Brent | 2008 | SIQ-Jr  Side Effects Form for Children and Adolescents. | SIQ-Jr: Baseline, 6 and 12 weeks.   Side-effects -weekly | 4 | 5.09 |
| DelBello | 2014 | Adverse events monitored | Various timepoints from screening onwards | 4 | 2.60 |
| De Jonge-Heesen | 2020 | Item 8 of CDI-2 | Screening and 6-month follow up | 2, 3 | 1.54 |
| Diamond | 2019 | SIQ-Jr | Baseline, 4, 8, 12, 16, 24, 32, 40, 52 weeks | 5 | 4.65 |
| Diamond | 2010 | SIQ-Jr | Weekly | 3 | 16.67 |
| Findling | 2020 | C-SSRS assessments and interview | During RCT and follow-up | 1 | 1.69 |
| Goodyer | 2004 | Risk and Self Harm Inventory and CSI | 36-, 52-, and 86-weeks post-randomisation | 4 | Recent suicide attempts: 3% at 36 weeks. 6% at 52 weeks.  NSSI attempts: 7% at 36 weeks, 4% at 52 weeks, and 5% at 86 weeks |
| Grudin | 2022 | MINI-KID and CDRS-R, Deliberate Self-Harm Inventory for youths | Baseline, follow up assessments, weekly throughout treatments | 2, 3 | NR |
| Herrera | 2025 | Modified C-SSRS | Baseline, days 4, 7/10, 30 and 60 | 4 | Baseline suicidal ideation: Over 90%  Day 60: 27% in experimental condition; 50% in control condition |
| Hughes | 2013 | QIDS-A | Weekly | 4 | NR |
| Iftene | 2015 | Adverse events monitored, item 9 of CDI | Baseline, 8 weeks, 16 weeks | 4 | 0 |
| Ip | 2016 | Usual measure - CEDS-R – recorded suicidal ideation | Pre-test, 4 months, 8 months, 12 months | 2 | NR |
| Keller | 2001 | Adverse events monitored | Weekly | 1 | 1.82 |
| Kosik-Gonzalez | 2025 | SIBAT | NR | 4 | 15.6 |
| Lan | 2023 | C-SSRS | Baseline, days 1, 2, 3, 4, 5, 6, 12 of trial, and at follow up | 1 | NR |
| Lindqvist | 2020 | C-SSRS | Screening and baseline | 2, 3 | NR |
| Liu | 2025 | SPS and MSHI | Baseline and post intervention | 4 | NR |
| March | 2004 | Yes - SIQ-Jr and harm-related adverse events monitored | Baseline, 6 and 12 weeks | 4 | 12.98 |
| McCauley | 2015 | Additional question added to the SMFQ | NR | 4 | NR |
| Nicol | 2022 | PHQ-9 | Baseline, 2,4,6,8,12 weeks | 2, 3 | 55.56 |
| O’Dea | 2020 | PHQ-9 | Baseline, 4 weeks, 12 weeks | 2 | NR |
| Pile | 2021 | MFQ Short with additional risk question | Baseline and beginning of each treatment session | 5 | 0 |
| Poppelaars | 2016 | Item 9 of the CDI | Baseline, weekly throughout intervention, post-test, 3, 6, 12 month follow ups | 2 | 2.88 |
| Ranney | 2018 | Crisis Management Protocol - disclosure of suicidal ideation | Throughout | 2 | NR |
| Saito | 2022 | Adverse events monitored, item 13 of the CDRS-R | Baseline, throughout study | 4 | 19.46 |
| Schniering | 2022 | Unspecified risk assessment | After randomisation to waitlist | 4 | NR |
| Shomaker | 2016 | K-SADS and programme content | Throughout study | 2 | NR |
| Wang | 2025 | BDI | NR | 5 | NR |
| Wilson | 2024 | Unspecified risk assessment | Baseline, 5, 10, 12 weeks | 2 | 12.50 |
| Wolff | 2020 | NR | During intervention | 3 | NR |
| Wright | 2017 | Modified MFQ | Baseline, treatment sessions, 4-month, 12-month follow up | 2 | 0 |
| Yang | 2016 | Unspecified measure | Baseline | 2 | 24 |
| Zsigo | 2023 | Unspecified measure | Throughout | 1 | 1.41 |

Codes:(1) discontinuation (referring to any studies that removed participants from the RCT when risk was reported); (2) onward referral or signposting (e.g., provision of crisis line contact details or referral to additional clinical services); (3) further clinical involvement from a member of the associated clinical team (e.g., study lead or clinical supervisor); (4) not recorded; and (5) other.

CDI- Children’s Depression Inventory, CDI-2 – Children’s Depression Inventory 2^nd^ Edition, CDRS-R- Children’s Depression Rating Scale- Revised, CEDS-R – Center for Epidemiologic Studies Depression Scale – Revised, CSI- Columbia Suicide Inventory, C-SSRS – Columbia-Suicide Severity Rating Scale, K-SADS – Kiddie Schedule for Affective Disorders and Schizophrenia, MFQ- Mood and Feelings Questionnaire, MINI-KID- Mini International Neuropsychiatric Interview for Children and Adolescents, MSHI- Modified Self-Harm Inventory, PHQ-9 – Patient Health Questionnaire-9, QIDS-A- The Quick Inventory of Depressive Symptomatology- Adolescent Version, RCT- Randomised Controlled Trial, SIBAT - Suicide Ideation and Behaviour Assessment Tool, SIQ-Jr – Suicidal Ideation Questionnaire- Junior, SMFQ- Short Mood and Feelings Questionnaire, SPS- Suicide Probability Scale, TEAE- Treatment-Emergent Adverse Event
