# Supplementary material for: How the risk of suicide and non-suicidal self-injury is assessed, monitored and managed in randomised controlled trials of interventions for youth depression: a scoping review
Source: BMJ Open. 2026 Apr 28;16(4):e111993. doi: 10.1136/bmjopen-2025-111993 (PMC13141124; doi:10.1136/bmjopen-2025-111993)
Supplement: Supplementary file 6 [file bmjopen-2025-111993.reviewer_comments.pdf]

PEER REVIEW HISTORY

BMJ Open publishes all reviews undertaken for accepted manuscripts. Reviewers are asked to complete a checklist review form and are provided with free text boxes to elaborate on their assessment. These free text comments are reproduced below.

ARTICLE DETAILS

Title (Provisional)

How Risk of Suicide and Non-Suicidal Self-Injury is Assessed, Monitored, and Managed in Randomised Controlled Trials of Interventions for Youth Depression: A Scoping Review

Authors

Hudson, Felicity; Read-Williams, Chloe; Williamson, Grace; Lobo, Samantha; Hardman, Amelia; Patel, Haleemah; Pile, Victoria

---

VERSION 1 - REVIEW

**Reviewer**	**1**
**Name**	**Ortiz-Prado, Esteban**
**Affiliation**	**Universidad de Las Américas**
**Date**	**12-Nov-2025**
**COI**	**None**

I appreciate the opportunity to review this manuscript. The topic is interesting and addresses a very specific and important gap in the literature. The idea of examining how risk of suicide and non-suicidal self-injury (NSSI) is handled in randomized controlled trials (RCTs) for adolescent depression is innovative and fills an important niche. Based on a brief search for similar works, while there are related systematic reviews on suicidality in depression trials, none appear to focus specifically on scoping the procedures for risk exclusion, monitoring, and management in youth populations. This underscores the need for your work.

That said, the title is somewhat confusing, as you noted. It could be clearer by emphasizing the focus on procedures rather than simply “assessing risk.” A suggested revision: “How Risk of Suicide and Non-Suicidal Self-Injury Is Assessed, Monitored, and Managed in Randomized Controlled Trials of Interventions for Youth Depression: A Scoping Review.” This better captures the procedural emphasis without losing key terms.

Limitations are acknowledged transparently, including the variability in risk definitions and the English-language restriction. The figures are clear but could be improved; the bar chart beside the pie chart seems unnecessary. Consider creating an infographic if possible; in my

view, pie charts are overly simplistic, but I defer to your judgment. Additionally, the discussion would benefit from addressing practical implications in broader global contexts where RCTs are uncommon and risk assessment is less robust. What are the potential benefits, and how might these procedures be adapted and scaled to resource-constrained settings, such as Latin America?

**Reviewer**	**2**
**Name**	**Tong, Yongsheng**
**Affiliation**	**Peking University, Huilongguan Clinical Medical School**
**Date**	**22-Nov-2025**
**COI**	**None**

I have no concerns on this interesting manuscript. Here is my comment.

Exclusion criteria of at high suicide risk is a double edged sword in any RCTs on treatment of mental disorders. Given suicidality is identified as a symptom of major depressive disorder (MDD), such criteria would result in many potential patients with MDD and high suicide risk being excluded from the RCT, which would also bring about that we do NOT know whether the excluded patients might benefit from the novel technique or medication. Meanwhile, the exclued patients need effective treatment urgently. That is, strict exclusion criteria might not always the best choice, although patients' safety is also one of the most important concerns in any RCTs.

**Reviewer**	**3**
**Name**	**Riblet, Natalie**
**Affiliation**	**Dartmouth College**
**Date**	**22-Nov-2025**
**COI**	**None**

This manuscript reports on a scoping review that explores how suicide risk is managed in RCTs of interventions to treat depression in young people. The manuscript makes a unique contribution to the literature by highlighting gaps in research including a lack of standardization of suicide risk/protocols in clinical trials and limited generalizability of results to those at risk for suicide. There are areas of the manuscript that require revision as outlined below.

General Comment

#1 I noticed that in the abstract as well as manuscript the term ‘risk’ is often used in isolation. This may confuse a reader. While I don’t believe that it is necessary to add

"suicide" to every sentence that mentions risk, I do feel that it would be helpful to remind the reader in a few places that the authors mean suicide risk rather than risk in general.

Background

#1 The authors have assimilated a lot of important information. That said, I found that the background was somewhat challenging to read. Several concepts were introduced throughout and it was not clear until I got to the last paragraph that the goal is to understand how researchers are managing suicide risk in clinical trials that enroll young people with depression. E.g., framing of the paragraphs in the existing background does not make it clear that the focus is on the context of assessing suicide risk in trials versus a non-research clinical setting.

#2 Related to point #1, I wonder if it may helpful to reframe the background. E.g., 1) Paragraph 1 – Focus first on prevalence of depression in youth (focus area of the scoping review) and discuss associated burdens in this group including high prevalence of comorbid SI/SB. 2) Paragraph 2 – Discuss that because effective treatments are needed to treat youth with depression, researchers conduct RCTs of pharmacological and behavioral interventions. In this context, however, there is a question about how researchers should effectively manage comorbid SI/SB risk. It's also possible that some researchers may decide to exclude patients because of these concerns, etc. 3) Paragraph 3- Introduce larger questions around risk assessments and harm definitions in the context of RCTs in youth with depression. Emphasize specific gaps in knowledge, 4) Paragraph 4- Discuss goals of the scoping review.

#3 If above changes are made, it will help the reader to understand the motivation for the 1st RQ of interest "Do RCTs for depression exclude young people based on risk?" In the current version, this first RQ seems to come out of the blue.

#4 There is a reference to global suicide rates and then specifically UK. This decision is not clear since it sounds like the focus of the review is global.

Results

#1 Recommend spelling out a number or percentage when it starts off a sentence.

#2 Under question 1, I think it may be helpful to have a subheading before 2nd paragraph so that it's clear that other paragraphs refer to those studies that had exclusion criteria.

#3 Did the authors include any studies whose research aim was actually to test an intervention in youth with depression and comorbid suicide risk? Given that there were a fair number of studies that excluded high-risk groups, it would be equally interesting to know whether any of the included studies were designed to test interventions to treat youth with depression and comorbid SI/SB (or deemed to be at high risk). One might imagine that the approach to risk management (and details outlined in the manuscript) may look quite different in studies that are overtly targeting a high-risk group.

#4 Related to point #3 it might be helpful in Table 1 to add a column that describes the target population for each of the studies (e.g., youth with MDD).

Discussion:

Overall, the discussion raises excellent points. Another question I wonder about is the issue of equity and whether this should be considered when researchers make the decision to exclude certain populations (such as those at high risk for suicide) from a study.

#1 Did the authors check to see whether the studies posted their protocol or other information to a database such as clinicaltrials.gov? This would be another place where authors may outline in greater detail how they assessed/managed risk even if they did not include it in their manuscript.

#2 Related to point #1, under limitations I recommend adding that the authors were working off of the data available in the manuscripts. Therefore, it's possible that certain steps may have occurred but they were simply not reported in the manuscript (applies to RQ 2 & 3).

Tables and Figures

#1 I recommend adding a descriptive header for each table. E.g., Table 1, clarify that this describes the baseline characteristics of RCTs of interventions for depression in youth.

#2 Table 1 and 2 – I wonder if an error occurred when Table 1 was uploaded. There appears to be a random "Ip" on a single line between Authors Iftene and Jones. Similarly, there appear to be some formatting issues in Table 2 (column for Year).

#3 Table 2 and 3 – I recommend seeing whether it's possible to add the exclusion reasons (table 2) and how risk was managed (Table 3) directly into the table rather than using a code (with legend). This makes it easier for the reader to quickly glance through the table.

General comment about writing style

#1 I recommend reviewing the manuscript. There were a few areas where the sentences could benefit from further editing to help with sentence flow (e.g., 2nd sentence under Assessment of methodological quality and publication bias).

#2 There are some places where more specification is needed in the sentence to help the reader understand what the sentence is referring to. E.g., "Risk in this context" (background) (?risk in what context, trials? Clinic, both?) or "There is ongoing debate…..in this population" (background) (which population do you mean, youth with depression?).

VERSION 1 - AUTHOR RESPONSE

Reviewer: 1
Dr. Esteban Ortiz-Prado, Universidad de Las Américas, Universitat de Barcelona
Comments to the Author:
I appreciate the opportunity to review this manuscript. The topic is interesting and addresses a very specific and important gap in the literature. The idea of examining how risk of suicide and non-suicidal self-injury (NSSI) is handled in randomized controlled trials (RCTs) for adolescent

depression is innovative and fills an important niche. Based on a brief search for similar works, while there are related systematic reviews on suicidality in depression trials, none appear to focus specifically on scoping the procedures for risk exclusion, monitoring, and management in youth populations. This underscores the need for your work.

Many thanks for your positive comments.

That said, the title is somewhat confusing, as you noted. It could be clearer by emphasizing the focus on procedures rather than simply "assessing risk." A suggested revision: "How Risk of Suicide and Non-Suicidal Self-Injury Is Assessed, Monitored, and Managed in Randomized Controlled Trials of Interventions for Youth Depression: A Scoping Review." This better captures the procedural emphasis without losing key terms.

Many thanks for this suggestion. We agree that this captures the procedural emphasis without losing key terms and have updated the title with your suggestion.
Title: How Risk of Suicide and Non-Suicidal Self-Injury is Assessed, Monitored, and Managed in Randomised Controlled Trials of Interventions for Youth Depression: A Scoping Review

Limitations are acknowledged transparently, including the variability in risk definitions and the English-language restriction. The figures are clear but could be improved; the bar chart beside the pie chart seems unnecessary. Consider creating an infographic if possible; in my view, pie charts are overly simplistic, but I defer to your judgment. Additionally, the discussion would benefit from addressing practical implications in broader global contexts where RCTs are uncommon and risk assessment is less robust. What are the potential benefits, and how might these procedures be adapted and scaled to resource-constrained settings, such as Latin America?

We have created an infographic to provide a visual summary of the key findings, please refer to the revised Figure 2.

Many thanks for highlighting the global implications of this work and the practical constraints. We have now highlighted this in the discussion.

Page 33: This review also highlights a significant disparity in this field of research between high- and low-income countries, with 92.13% of the studies conducted in high income countries. Globally, RCTs remain less common in low-income countries. Beyond the need to understand how research can be effectively delivered in more resource-constrained environments, it is essential to critically examine how risk assessments can be strengthened across diverse cultural and socioeconomic contexts. Furthermore, current procedures should be evaluated and adapted to ensure that they are contextually appropriate and feasible within these settings.

Reviewer: 2
Dr. Yongsheng Tong, Peking University
Comments to the Author:
I have no concerns on this interesting manuscript. Here is my comment.

Exclusion criteria of at high suicide risk is a double-edged sword in any RCTs on treatment of mental disorders. Given suicidality is identified as a symptom of major depressive disorder (MDD), such criteria would result in many potential patients with MDD and high suicide risk being excluded from the RCT, which would also bring about that we do NOT know whether the excluded patients might benefit from the novel technique or medication. Meanwhile, the excluded patients need effective treatment urgently. That is, strict exclusion criteria might not always the best choice, although patients' safety is also one of the most important concerns in any RCTs.

Many thanks for this thoughtful comment, we fully agree about this tension between ethical and clinical decision making in the methodology for RCTs. We have expanded our dialogue on this in

both the introduction and discussion. We hope the paper highlights this dilemma that many researchers face and offers reflections on possible future directions.

Reviewer: 3
Dr. Natalie Riblet, Dartmouth College
Comments to the Author:
This manuscript reports on a scoping review that explores how suicide risk is managed in RCTs of interventions to treat depression in young people. The manuscript makes a unique contribution to the literature by highlighting gaps in research including a lack of standardization of suicide risk/protocols in clinical trials and limited generalizability of results to those at risk for suicide. There are areas of the manuscript that require revision as outlined below.

General Comment
#1 I noticed that in the abstract as well as manuscript the term 'risk' is often used in isolation. This may confuse a reader. While I don't believe that it is necessary to add "suicide" to every sentence that mentions risk, I do feel that it would be helpful to remind the reader in a few places that the authors mean suicide risk rather than risk in general.

Many thanks for highlighting this, we have now added several mentions of "suicide" throughout the manuscript to remind the reader that this is what we are referring to. This includes three additions of *"*risk of suicide or self-harm*"* in the abstract and sixteen similar mentions throughout the manuscript.

Background
#1 The authors have assimilated a lot of important information. That said, I found that the background was somewhat challenging to read. Several concepts were introduced throughout and it was not clear until I got to the last paragraph that the goal is to understand how researchers are managing suicide risk in clinical trials that enroll young people with depression. E.g., framing of the paragraphs in the existing background does not make it clear that the focus is on the context of assessing suicide risk in trials versus a non-research clinical setting.

We have now significantly restructured the introduction. We believe that the purpose of the review is now much clearer. Given that these are changes are extensive, we have not pasted them below but we have included a tracked change version of the manuscript.

#2 Related to point #1, I wonder if it may helpful to reframe the background. E.g., 1) Paragraph 1 – Focus first on prevalence of depression in youth (focus area of the scoping review) and discuss associated burdens in this group including high prevalence of comorbid SI/SB. 2) Paragraph 2 – Discuss that because effective treatments are needed to treat youth with depression, researchers conduct RCTs of pharmacological and behavioral interventions. In this context, however, there is a question about how researchers should effectively manage comorbid SI/SB risk. It's also possible that some researchers may decide to exclude patients because of these concerns, etc. 3) Paragraph 3- Introduce larger questions around risk assessments and harm definitions in the context of RCTs in youth with depression. Emphasize specific gaps in knowledge, 4) Paragraph 4- Discuss goals of the scoping review.

Thank you, we have now restructured the introduction following this guidance as much as possible. The first paragraph focuses on the prevalence of suicidality and self-harm and its relationships with depression. We then focus on the need for effective interventions and the current landscape, and from here discuss the challenges of managing comorbid risk of suicide or self-harm in this population. This helps to focus the third section of the introduction and frame the questions around risk assessments, how risk is defined, and how it is managed in trials. This structure results in a much clearer lead into the goals of the scoping review in the final section and the motivation behind each research question. Given the extent of these revisions, we have included them here.

#3 If above changes are made, it will help the reader to understand the motivation for the 1st RQ of interest "Do RCTs for depression exclude young people based on risk?" In the current version, this first RQ seems to come out of the blue.

Following our restructure as per your above recommendation, we believe RQ1 now logically leads on from the introduction.

#4 There is a reference to global suicide rates and then specifically UK. This decision is not clear since it sounds like the focus of the review is global.

The review includes all studies that evaluate an intervention for depression and we agree that the reference to the UK is misleading and so we have removed this sentence from the introduction.

Results

#1 Recommend spelling out a number or percentage when it starts off a sentence.

We have now amended these sentences and spelt out any numbers or percentages that are at the start of sentences. For example:
Page 23: Twelve studies signposted participants to other services or directly referred participants for further support.

#2 Under question 1, I think it may be helpful to have a subheading before 2nd paragraph so that it's clear that other paragraphs refer to those studies that had exclusion criteria.

We have now added a subheading beneath the initial result of how many studies excluded for risk to indicate that the rest of that research question refers only to those with exclusion criteria.
Page 17: A total of 49 (55.06%) studies excluded young people either at the screening or baseline phase for risk. The other studies did not specify risk in their exclusion criteria or in the study procedure.
Studies excluding participants for risk of suicide or self-harm

#3 Did the authors include any studies whose research aim was actually to test an intervention in youth with depression and comorbid suicide risk? Given that there were a fair number of studies that excluded high-risk groups, it would be equally interesting to know whether any of the included studies were designed to test interventions to treat youth with depression and comorbid SI/SB (or deemed to be at high risk). One might imagine that the approach to risk management (and details outlined in the manuscript) may look quite different in studies that are overtly targeting a high-risk group.

Thank you for noting this distinction in the study aims. Three studies were aimed at a population with suicide risk and so we have added this into the discussion to reflect the different ways that these studies might manage risk.
Page 29: This distinction can be seen in the three studies which were specifically targeting adolescents with suicidal ideation or intent. Two of the studies [39, 61] did not exclude any participants based on risk but instead specified the risk management procedures in place, whilst the third [38] specified an exclusion of those at imminent risk that could not be safely treated on an outpatient basis due to the limitations of the intervention.

#4 Related to point #3 it might be helpful in Table 1 to add a column that describes the target population for each of the studies (e.g., youth with MDD).

Thank you for this suggestion. This column has now been added, please see **Table 1**.

Discussion:

Overall, the discussion raises excellent points. Another question I wonder about is the issue of equity

and whether this should be considered when researchers make the decision to exclude certain populations (such as those at high risk for suicide) from a study.

#1 Did the authors check to see whether the studies posted their protocol or other information to a database such as clinicaltrials.gov? This would be another place where authors may outline in greater detail how they assessed/managed risk even if they did not include it in their manuscript.
#2 Related to point #1, under limitations I recommend adding that the authors were working off of the data available in the manuscripts. Therefore, it's possible that certain steps may have occurred but they were simply not reported in the manuscript (applies to RQ 2 & 3)

Thank you for this suggestion. We did not check databases for each study. The design of this study was a scoping review in which we intended to capture a large number of journal articles and understand the published literature. We have now included this as a limitation of our review, as recommended.

Page 32: Third, the review assessed studies based solely on published manuscript data without checking for registered protocols. Consequently, some risk assessment steps may have been undertaken and reported in online databases but not reported in the final publications.

Tables and Figures
#1 I recommend adding a descriptive header for each table. E.g., Table 1, clarify that this describes the baseline characteristics of RCTs of interventions for depression in youth.
Thank you for identifying this, each of the tables now has a descriptive header.

Page 11: Table 1. Characteristics of RCTs of interventions for depression in youth
Page 19: Table 2. Reasons and methods for excluding for risk in RCTs of interventions for depression in youth
Page 25: Table 3. Methods of monitoring risk in RCTs of interventions for depression in youth

In addition, we have added Figure headings into the manuscript, with suggested placeholders:

Page 9: [SUGGESTED POSITION FOR FIGURE 1]
Figure 1: Study Selection Process (PRISMA Diagram)
Page 22: [SUGGESTED POSITION FOR FIGURE 2]
Figure 2: Summary of key findings

#2 Table 1 and 2 – I wonder if an error occurred when Table 1 was uploaded. There appears to be a random "Ip" on a single line between Authors Iftene and Jones. Similarly, there appear to be some formatting issues in Table 2 (column for Year).

We have now corrected the tables in order to improve readability and check for mistakes. In the case of "Ip", this is the author of one of the papers, but the formatting misaligned the authorship with the results pertaining the study.

#3 Table 2 and 3 – I recommend seeing whether it's possible to add the exclusion reasons (table 2) and how risk was managed (Table 3) directly into the table rather than using a code (with legend). This makes it easier for the reader to quickly glance through the table.

Thank you for this helpful suggestion. We carefully explored incorporating the exclusion reasons (**Table 2**) and risk management details (**Table 3**) directly into the tables rather than presenting them as coded entries with a legend. However, doing so substantially increased the length and density of the tables, which we felt reduced clarity and made it more difficult for readers to quickly synthesise the key information. To preserve readability while maintaining transparency, we have retained the coded format in the main tables. We would be very happy to provide expanded versions of Tables 2 and 3,

with the full exclusion reasons and risk management details written out, as supplementary material if the reviewer feels this would be helpful.

General comment about writing style
#1 I recommend reviewing the manuscript. There were a few areas where the sentences could benefit from further editing to help with sentence flow (e.g., 2nd sentence under Assessment of methodological quality and publication bias).

We have now reviewed the manuscript carefully and edited to improve sentence flow. For example:
Page 8: This manuscript was prepared with reference to the PRISMA-ScR reporting guidelines and checklist (please refer to supplementary material C).[23]

#2 There are some places where more specification is needed in the sentence to help the reader understand what the sentence is refergo
ring to. E.g., "Risk in this context" (background) (?risk in what context, trials? Clinic, both?) or "There is ongoing debate.....in this population" (background) (which population do you mean, youth with depression?).

We have reviewed the manuscript and added more specification to aid readability. For example:

Page 5: Currently, there is considerable variability in the ways that risk is both defined and monitored across research trials.[13] Risk in this context can be defined as the possibility of harm, i.e. the suggestion that something may occur without implying that it is probable or will occur.[14]

Page 5: Another approach is to use standardised risk assessments but there is ongoing debate about their clinical utility, with concerns about the tools currently available.[17]

VERSION 2 - REVIEW

**Reviewer**	**3**
**Name**	**Riblet, Natalie**
**Affiliation**	**Dartmouth College**
**Date**	**26-Mar-2026**
**COI**	

The authors did an excellent job responding to my critique. This scoping review is an important contribution to the literature.
